# The determinants of effective inter-organization information sharing in the health capital planning process

**DOI:** 10.1177/08404704221087408

**Published:** 2022-05-04

**Authors:** Rayeh Kashef Al-Ghetaa, Imtiaz Daniel, James Shaw, David Klein, Adalsteinn Brown

**Affiliations:** 1 3710 McMaster University, Hamilton, Ontario, Canada.; 2 7938 University of Toronto, Toronto, Ontario, Canada.

## Abstract

This qualitative study examines the determinants of effective inter-organization information sharing in the Health Capital Planning process (the process), primarily in the final stage of the process which focuses on the review of final expenses and release of a holdback. Using thematic analysis and building off a scoping review that was conducted in preparation for this study, we provide a framework for effective information sharing during the process. We interviewed 17 leaders from the Government of Ontario and hospitals across the province. The results of the interviews indicate that the most essential determinants of effective inter-organization information sharing in the process: organizational characteristics; reducing complex bureaucracies; preserving human resources and expertise; clear and standardized information; reducing policy changes; networks; negotiation abilities; information technology; training; record retention; and early planning. This study confirmed the need for effective intra-organization and interpersonal information sharing to achieve successful inter-organization information sharing.

## Introduction

Capital represents the total funds allocated and spent by a Health Service Provider (HSP) to build, acquire, or renovate health physical assets such as property, buildings, technology, or equipment.^1^ In Ontario, Canada, health capital planning and oversight is the responsibility of the provincial government.^2^ The Ontario Health Capital Planning process (the process) consists of 5 major stages with the final funding approval sub-stage known as the Final Cost Reconciliation (final) stage.^3,4^ This stage begins when the project is 100% complete around ten years after Stage 1 of the project.^3,4^ During this stage, HSPs provide their audited expenses for assessment by Ministry of Health (MOH) for the release of the remaining balance of their health capital funds. The balance is 5% of the total project cost.^3^ Every stage of the process relies heavily on the previous one and requires regulatory and often funding approval from the MOH.^4^ During the final stage, all partners involved must have a clear understanding of not only the requirements of the stage itself but also the full process and a detailed history of the project.^4^

Infrastructure planning is complex and lengthy in any sector and in any jurisdiction.^5,6^ Given that, effective information sharing is critical but challenging.^5,6^ This qualitative study investigates the determinants of effective inter-organization information sharing in health capital planning by examining the Ontario process from the perspective of MOH leaders, the Ministry of Infrastructure (MOI), and hospitals.

## Theory

Infrastructure planning remains understudied in the literature^1,2,7^ particularly within the realm information sharing.^5,6^ When an infrastructure project is initiated, numerous partners must collaborate to deliver the project.^8^ Throughout the multi-phased life cycle of a health capital project (such as planning, tendering, and construction), a large amount of information is produced on project scope, budget, risk, and approvals.^5^ Therefore, effective information retention and sharing between the partners involved is crucial.^5,6^ We chose to focus on the Final Cost Reconciliation stage since it is the last stage of the process in Ontario and requires the partners of the project to have all the necessary information to be able to reconcile audited costs against the estimated costs of the project.^3^

This qualitative study builds on two previous studies. A literature review on the factors influencing information sharing^9^ and an unpublished scoping review that was conducted in preparation for this paper. Yang and Maxwell reviewed existing literature to identify factors most critical for successful information sharing within public organizations and establish three independent information sharing frameworks: interpersonal, intra-organizational, and inter-organizational.^9^

The modified framework that we generated through our scoping review confirmed the importance of the elements that Yang and Maxwell^9^ outlined and built on them by identifying further determinants of effective inter-organization information sharing specifically for the planning of publicly funded infrastructure projects:^8,10-48^ frequency of communication; alignment of goals; contracts and record management; clarity; and reducing information asymmetry and clarity. Our framework demonstrated that it is challenging for organizations to implement effective inter-organization information sharing without first implementing effective interpersonal and intra-organization information sharing. This means that to ensure effective inter-organization information sharing when planning infrastructure projects, all three frameworks that Yang and Maxwell identified should be combined and the additional factors we identified should be built into the framework. The framework we established through the scoping review informed the development of the research question, the interview guide for this study, and served as the basis for the framework we established through this study.

## Material and methods

With ethics approval from the University of Toronto Health Sciences Research Ethics Board, and informed consent from participants, we conducted semi-structured interviews from November 1, 2020 to March 17, 2021 with members of the Senior Management Group (SMG) using purposeful sampling and non-SMG individuals using snowball sampling from (a) MOH, (b) MOI, and (c) hospitals across the province. For this paper, SMG individuals are those with executive level roles. Participants with knowledge of the final stage of the Ontario process were included. Data collection ended when the dataset engendered sufficient information power to draw meaningful conclusions in response to the research questions.^49^ The data were analyzed using inductive and deductive thematic analysis^50^ (Supplemental material 1^51^).

## Results

We spoke to participants about their perception on the determinants of effective information sharing in Ontario’s process, particularly the final stage. The views were consistent from the hospitals’ perspective and the government of Ontario’s perspective. Based on this study’s findings, we designed a framework summarizing the determinants of effective information sharing in health capital planning (Tables 1 and 2).

**Table 1.** Description of the study participants.

*Participants' characteristics (*N *= 16)*	*Sum*	*%*
*Sector*		
Health service provider	11	69%
Government body	6	38%
*Region represented*		
North	1	6%
South	3	19%
East	4	25%
Greater Toronto area (GTA)	4	25%
All	5	31%
*Role*		
Senior management group	8	50%
Non-senior management group	9	56%
*Years of experience in infrastructure planning*		
Less than 5 years	2	13%
5 to 10 years	6	38%
Over 15 years	9	56%

**Table 2.** Participants' experience with information sharing during the financial reconciliation stage.

Category	Quotation
*Challenges*	
a) Different organizational characteristics	“When there's a three-way partnership, it leads to a potential break down in information” (Participant 11)
b) Complex bureaucracy	“The capital planning process is not actually 6 steps, it is more like 14 because we have step 1A, step 1B, step 2A, and step 2B” (Participant 11)
c) Human resources and expertise	“We have projects outstanding and obviously everybody’s left. You cannot find the documentation because of the turnover. It is also fairly complex, so it is not something someone can just pick up off the shelf and execute. And MOH was asking questions that we just could not answer or did not have the supporting documentation to answer” (Participant 13) “The stage requires “a certain amount of experience, history of the project and specific skills that are not easily teachable to a new staff” (Participant 6)
d) Lack of a clear and standardized information	“There is a process. But I’m not entirely sure just how well known the process is to the sector” (Participant 6) “There needs to be very clear communication with respect to what kind of documentation we need to keep and for how long” (Participant 5)
e) Policy changes	“Staff have to continuously adapt, learn and consistently apply the new policy” (Participant 15)
*Resources*	
a) Networks	“Having those touch points throughout the construction period too, to say look, you need to include this detail and change orders…So if the staff does turnover it is like not everything is completely lost and we have to start from scratch essentially” (Participant 8)
b) Negotiation abilities	“We get into a bit of a negotiation around things that are unknown conditions because, although they say that the architect’s responsible to review everything and know about everything that’s knowable beforehand, everybody knows that is not possible” (Participant 2)
c) Information technology advancement	“Information retention is getting easier, because I do not know about other hospitals, but we are fully electronic now” (Participant 13)
d) Forms and guidelines	“The cost-share guide which outlines the rules around shareable and non-shareable costs by the Ministry; the capital planning manual which outlines MOH’s requirements for the planning and approval of health capital project; and finally, the Final Cost Reconciliation template” (Participant 8)
e) Training	“We do have a settlement presentation now... I would say that has been around for a couple years now” (Participant 8).
*Recommendations*	
a) Early planning	“If there is staff turnover or there were issues with data quality, then we could address them right away, rather than wait for the reconciliation stage which could be a number of years” (Participant 8)
b) Effective record-keeping	“You are in construction and you see something that you think might be controversial, take photographs or you have records” (Participant 2)
c) Ensure clear and standardized information	"I do think from the information flow perspective, ensuring standardization and educating the sector on the process are required” (Participant 11)
d) Reduce complex bureaucracies	“We built it in 2013 and I think we took occupancy in 2015 or started the program in 2015 and we had it settled by 2018… and we had projects were ‘03 to ‘07 type timeframe and we were still reconciling them up until 2017 or 2018” (Participant 4)
e) Preserve human resources and expertise	“Hospitals that have more capital projects tend to be a bit more familiar or savvy with what is allowable in terms of expenditures and what is not” (Participant 10)

### Challenges

One of the leading reasons for the challenges associated with the stage is different organizational characteristics (a). The process requires at least 2, and in some cases 3, different organizations with different rules and procedures to collaborate and coordinate the delivery of a complex project.

The second leading challenge is navigating complex bureaucracies (b), which is a result of the length and complexity of the process in general. All stages of the process are mandatory for every project regardless of the size, scope, and magnitude.

Due to the length of the process, most organizations are unable to retain the human resources and expertise (c) needed to fulfill the requirements of the stage. Staff turnover means that “a lot of the corporate memory has moved on” (Participant 7). Therefore, organizations struggle with their ability to complete and support the requirements of the stage.

Participants indicated that the lack of a clear and standardized process (d) adds another layer of complexity to the stage. Participants from both MOH and hospitals acknowledged that the process does exist, but it is just not very well communicated and understood by the sector. The “timelines and expectations” on when or who should initiate the stage should be explained to the sector (Participant 6). Hospitals are not clear on the documentation they need to maintain throughout the cycle of their health capital projects.

Finally, participants also confirmed that policy changes (e) happen regularly, which has an impact on the progress of work of both the hospitals and MOH. Given that the stage is the final funding sub-stage of the process, and it relies heavily on the previous stages of the process, any change in policy at any stage can be challenging for organizations when reconciling a project.

### Resources available

Most participants indicated there are strong networks (a) and collaboration between the hospitals and MOH when it comes to the final stage. Participants confirmed that regular communications between MOH and HSPs throughout the process have a positive impact on the efficiency of the stage

Hospitals confirmed that they can negotiate (b) with MOH if they disagree with the Ministry’s assessment of their submission. The negotiations are around change orders. Hospitals can provide additional supporting documents if they believe an item should be funded.

Participants highlighted that although still challenging, record retention became easier with infrastructure technology advancement (c). In terms of supporting material and templates, MOH provides three essential forms and guidelines document (d). Finally, in the recent years, MOH introduced a training presentation (e) that can be shared with HSPs.

### Recommendations

Participants indicated that early planning (a) is crucial. It is important to ensure that all parties involved in the project understand and agree on the scope and cost of the project from the beginning of the process. If there are changes to the scope of the project (“change order”) which are common with construction projects, participants recommended assessing them immediately and discussing them with MOH to avoid loss of knowledge at the end of the process.

Participants confirmed the importance of record-keeping (b) throughout the process to ensure knowledge retention and mitigate the issue of staff turnover. Hospitals indicated that having clear and standardized information (c) around timelines, who should initiate the process, and guidelines from MOH would make the stage more efficient and encourage the organization to “act on reconciling their projects” (Participant 1). There needs to be clarity around what is required in terms of documentation as there are costs associated with record retention from the hospitals’ side. Participants, especially from the hospitals indicated that some of the guiding documents and templates provided by MOH should be enhanced. Many of them were not aware of the presence of the training document and have indicated that submission templates are difficult to use and “have limited instructions.” (Participant 1)

**Figure 1.**
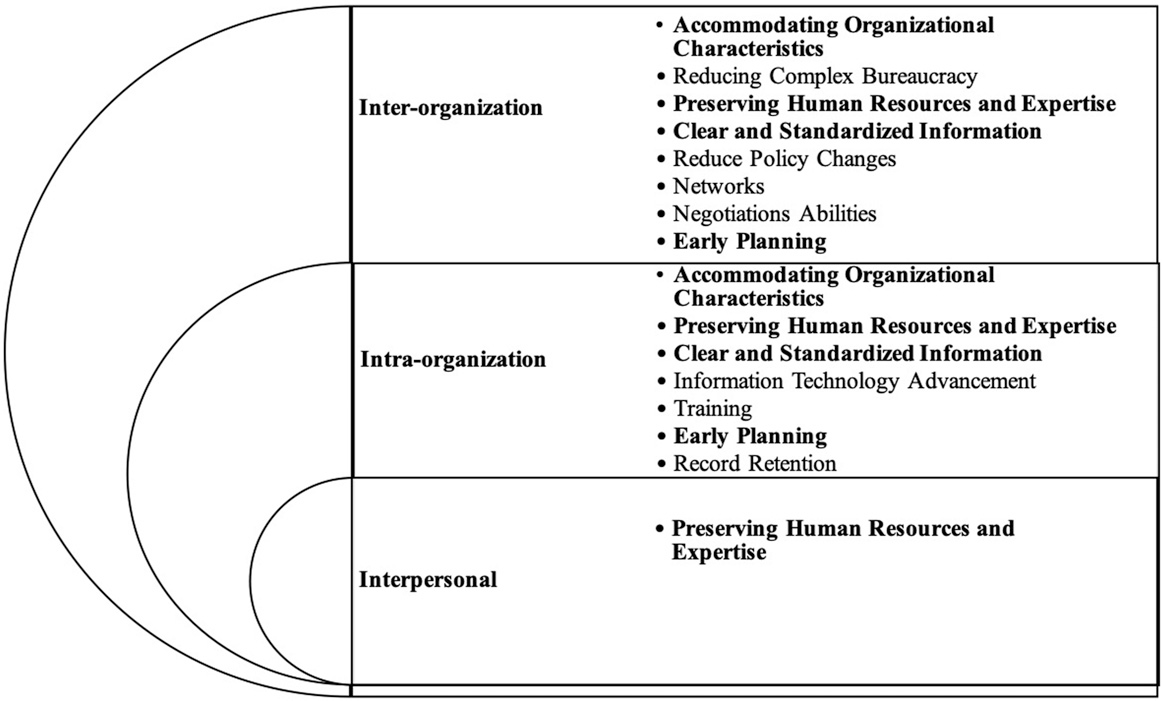
Summarizes the factors most essential for effective information-sharing in health capital planning. The figure also demonstrates the need for effective intra-organizational and interpersonal information sharing to achieve successful inter-organization information sharing in the planning of infrastructure projects.

Participants from MOH indicated that they have been working on streamlining the requirements of the stage to reduce complex bureaucracies (d). Hospitals indicated that recently the final stage became faster and more efficient. Finally, preserving human resources and expertise (e) remains a top priority for organizations when it comes to the final stage. Due to the technical nature of the stage, participants confirmed the importance of maintaining the knowledge and experience of their staff (Figure 1).

## Discussion

We examined the determinants of effective inter-organization information sharing in health capital planning by examining the Ontario process, specifically its final stage. Through the interviews conducted for this study, most of the participants indicated that the planning and funding of a health infrastructure project require extensive collaboration and coordination enabled by effective information sharing within the organizations involved and between them.

Participants emphasized the importance of staff and knowledge retention to reduce delays that can result from training new individuals.^40^ The importance of technical expertise was highlighted throughout the interviews.^7,27^ This will help partnering organizations ensure strong oversight of their projects, reduce the margin of error, and the need for change orders.

Our study also confirmed importance of certain determinants of effective inter-organization information sharing that already exist in the literature: clear instructions, standardized information, and the simplification of the overall capital planning process.^22,9^ Participants indicated that confusion around the process, especially in terms of roles and responsibilities, timelines, changing policies, and limited guidelines, can be restraining when it comes to prioritizing and completing the requirements of the process. Participants also confirmed that record-keeping is important to be able to respond to clarifications on expense items, and to effectively negotiate when there is a disagreement on the funding eligibility for projects.^20^

Finally, we confirmed the importance of working closely with all the contributors to a health capital project early in the planning process to ensure that all the parties understand and agree on the goals of the project including, scope and cost. This will reduce the potential for conflict between contributors, limit change orders, and lead to effective negotiations.

### Implications and future direction

Our study outlines to experts the challenges, resources, and importance of determinants that influence the effectiveness of inter-organization information sharing in planning complex public projects such as capital. In terms of practical implementation of our findings: first, policy-makers could benefit from examining the challenges that we outline in this paper to understand the barriers that planners experience. Second, the planners such as HSPs could benefit from the resources available to them as outlined in our paper. Our paper found that some resources are not well advertised to the sector. For example, there is a training document that MOH developed that most of our participants were not aware of it. Finally, both policy-makers and planners could benefit from our recommendations. Our study suggests that organizations should review the quality of their inter-organization information sharing enablers against our recommendations to identify areas of improvement and set up strategies to enhance their practices and the capital planning process. For example, our framework suggests that staff retention and technical expertise play an important role in enabling effective inter-organization information sharing in the health capital planning process. Organizations could benefit from evaluating their current staff and knowledge retention trends and put in place strategies to preserve them.

On the research side, there continues to be a need for studies on improving the efficiency of health capital planning.^1,2,7^ Our study contributes to the literature of efficient infrastructure planning and effective inter-organization information sharing. We confirmed the importance of the factors most critical to effective information sharing as established in existing literature,^8-48^ outlined a new factor (early planning), and produced an inter-organization information sharing framework to guide health capital planning. Our study confirmed that for organizations to successfully collaborate to plan and manage a health capital project, the three information sharing frameworks that Yang and Maxwell identified^9^ must build off each other. We showed that for organizations managing publicly funded capital projects, it is essential to ensure effective flow of information from within and between the organizations. This is likely due to the nature of the projects which requires collaboration and coordination across different levels of authority and the involvement of a wide range of technical expertise.

Further research is required to understand the fiscal and opportunity cost of improving the effectiveness of inter-organization information sharing in the process. We recommend more research to determine if further delays and inefficiencies can result from organizations investing their efforts into ensuring effective inter-organization information sharing in the health capital planning. Finally, future research is needed to examine the utility of our framework in other jurisdictions where policy-makers either control oversee or are responsible for enabling and facilitating the capital raising or expenditure for large public institutions.

### Limitations

First, given the technical and specialized nature of the process, we selected our study participants using a mix of purposeful and snowball sampling methods, which in turn could mean that our findings may not be representative of the entire health capital sector in Ontario. However, to help alleviate the selection bias associated with our study, we interviewed participants until the dataset engendered sufficient information power to draw meaningful conclusions.^10^

Second, the focus of the study is on health capital planning, and at first glance, the findings may not seem extendable to other public sectors or to non-public, market-driven fields. However, we would argue that understanding the challenges associated with inter-organization information sharing and the mitigation strategies that our study offers can be beneficial to any field, especially those managing large complex projects.

Finally, the focus of our study is on publicly funded projects and may not seem applicable to private infrastructure developments. However, we argue although our findings geared more towards optimizing public resources, they offer applicable recommendations to privately funded projects as well. Ultimately our study looks at the determinants of effective flow of information between funders and service providers to optimize the available resources. Therefore, despite the specific context of the study, the recommendations can be beneficial and transferrable to different sectors.

## Conclusion

Through the interviews conducted for this study, most of the participants indicated that the planning and funding of a health infrastructure project require extensive collaboration and coordination enabled by effective information sharing within the organizations involved and between them. This is particularly true in the case of the final stage of the Ontario process, which requires extensive intra-organization and inter-organization work. Therefore, we chose to investigate the Ontario process to identify the determinants of effective inter-organization information sharing in the planning of public health capital projects.

## References

[1] Klein DJ Brown AD Detsky AS . Investing wisely in health care capital. JAMA. 2016;316(15):1543-1544. doi:10.1001/jama.2016.10605. 27684645

[2] Brown AD Klein DJ Huynh TM . Capital Spending in Healthcare: A Missed Opportunity for Improvement?; 2013.

[3] Ministry of Health . Capital Planning Manual; 1996. https://www.google.com/search?q=mohltc+capital+planning+manual&oq=MOHLTC+ca&aqs=chrome.1.69i57j35i39l2j0j0i22i30l6.2640j1j7&sourceid=chrome&ie=UTF-8 (Accessed April 27, 2021).

[4] Decter M . Ontario’s Health Capital Planning Review; 2003. https://news.ontario.ca/en/release/92868/ontario-government-releases-report-on-ontarios-health-capital-planning-review (Accessed April 27, 2021).

[5] Zhang L Yuan J Xia N Ahmed B Al-Hussein M Asce SM . Improving Information Sharing in Major Construction Projects through OC and POC: RDT Perspective; 2020. Published online. 10.1061/(ASCE)CO.1943-7862.0001847.

[6] Collinge W Harty C Liu K Tang Y . Improving information sharing across construction stakeholders: an organizational semiotics approach. In: Joint international symposium 2009, Dubrovnik, Croatia, 2009. https://www.researchgate.net/publication/265849140_Improving_information_sharing_across_construction_stakeholders_an_organizational_semiotics_approach (Accessed April 12, 2021).

[7] Teja B Daniel I Pink GH Brown A Klein DJ . Ensuring adequate capital investment in canadian health care. CMAJ. 2020;192(25):E677-E683. doi:10.1503/cmaj.191126. 32571884 PMC7828857

[8] Gamper C Charbit C . Coordination of infrastructure investment across levels of government. 2014. https://scholarworks.gsu.edu/icepp/16. https://scholarworks.gsu.edu/icepp/16 (Accessed December 23, 2020).

[9] Yang TM Maxwell TA . Information-sharing in public organizations: a literature review of interpersonal, intra-organizational and inter-organizational success factors. Gov Inf Q. 2011;28(2):164-175. doi:10.1016/j.giq.2010.06.008.

[10] Agranoff R . MANAGING COLLABORATIVE PERFORMANCE: changing the boundaries of the state? Public Perform Manag Rev. 2014;29(1):18-45. https://www.tandfonline.com/doi/abs/10.1080/15309576.2005.11051856 (Accessed December 23, 2020).

[11] Ahmad E Bhattacharya A Vinella A Xiao K . Involving the private sector and public-private partnerships in financing investments: public opportunities and challenges. 2015. https://greengrowthknowledge.org/research/involving-private-sector-and-public-private-partnerships-financing-investments-public. https://greengrowthknowledge.org/research/involving-private-sector-and-public-private-partnerships-financing-investments-public (Accessed December 23, 2020).

[12] Akinkugbe OD . The dilemma of public-private partnerships as a vehicle for the provision of regional transport infrastructure development in Africa. Law Dev Rev. 2013;6(2):3-27. doi:10.1515/ldr-2013-0018.

[13] Aluonzi G N Oluka P Nduhura A . Contract management and performance of road maintenance projects: the case of arua municipality. Universal Journal of Management. 2016;4(10):550-558. doi:10.13189/ujm.2016.041004.

[14] Amirkhanyan AA . Collaborative performance measurement: examining and explaining the prevalence of collaboration in state and local government contracts. Journal of Public Administration Research and Theory. 2009;19(3):523-554. doi:10.1093/jopart/mun022.

[15] Aslan C Duarte D . How Do Countries Measure, Manage, and Monitor Fiscal Risks Generated by Public-Private Partnerships? Chile, Peru, South Africa, Turkey: The World Bank; 2014. doi:10.1596/1813-9450-7041.

[16] Bakvis H Browny D . Policy coordination in federal systems: comparing intergovernmental processes and outcomes in Canada and the United States. Publius. 2010;40(3):484-507. doi:10.1093/publius/pjq011.

[17] Becker F Patterson V . Public: private partnerships: balancing financial returns, risks, and roles of the partners. Public Perform Manag Rev. 2005;29(2):125-144. https://www.jstor.org/stable/20447583?seq=1 (Accessed December 23, 2020).

[18] Berg Sv Horrall J . Networks of regulatory agencies as regional public goods: improving infrastructure performance. Rev Int Org. 2008;3(2):179-200. doi:10.1007/s11558-007-9028-8.

[19] Bloomfield P . The challenging business of long-term public-private partnerships: Reflections on local experience. Public Adm Rev. 2006;66(3):400-411. doi:10.1111/j.1540-6210.2006.00597.x.

[20] Carbonara N Pellegrino R . Fostering innovation in public procurement through public private partnerships. J Public Procure. 2018;18(3):257-280. doi:10.1108/JOPP-09-2018-016.

[21] Cerra V Cuevas A Goes C , et al. Highways to heaven: infrastructure determinants and trends in Latin America and the Caribbean. J Infrastruct Policy Devt. 2017;1(2):168-189. https://www.imf.org/en/Publications/WP/Issues/2016/12/31/Highways-to-Heaven-Infrastructure-Determinants-and-Trends-in-Latin-America-and-the-Caribbean-44272 (Accessed December 23, 2020).

[22] Dawes SS . Interagency information sharing: expected benefits, manageable risks. Journal of Policy Analysis and Management. 1996;15(3):377-394. doi:10.1002/(SICI)1520-6688(199622)15:3<377::AID-PAM3>3.0.CO;2-F.

[23] de Palma A Leruth L Prunier G . Towards a principal-agent based typology of risks in public-private partnerships. Reflets et perspectives de la vie économique. 2012;LI(2):57. doi:10.3917/rpve.512.0057.

[24] Dulaimi MF Ling FYY Bajracharya A . Organizational motivation and inter-organizational interaction in construction innovation in Singapore. Constr Manag Econ. 2003;21(3):307-318. doi:10.1080/0144619032000056144.

[25] Forrer J Kee JE Newcomer KE Boyer E . Public-private partnerships and the public accountability question. Public Adm Rev. 2010;70(3):475-484. doi:10.1111/j.1540-6210.2010.02161.x.

[26] Giorno C . Meeting infrastructure needs in Australia; 2011. doi:10.1787/5kgg7sx3p7q0-en.

[27] Gomes CF Small MH Yasin MM . Towards excellence in managing the public-sector project cycle: a TQM context. Int J Public Sector Manag. 2019;32(2):207-228. doi:10.1108/IJPSM-11-2017-0315.

[28] Han C . Trilateral cooperation with China | UNDP in China. 2016. https://www.cn.undp.org/content/china/en/home/library/south-south-cooperation/trilateral-cooperation-with-china-.html. https://www.cn.undp.org/content/china/en/home/library/south-south-cooperation/trilateral-cooperation-with-china-.html (Accessed December 23, 2020).

[29] Helm D . British infrastructure policy and the gradual return of the state. Oxf Rev Econ Policy. 2013;29(2):287-306. doi:10.1093/oxrep/grt018.

[30] Hilvert C Swindell D . Collaborative service delivery: what every local government manager should know. State Local Gov Rev. 2013;45(4):240-254. doi:10.1177/0160323x13513908.

[31] Ibrahim AD Price A Khalfan MMA Dainty A . Construction procurement strategies of national health service in the UK: a critical review. J Public Procure. 2010;10(1):31-67. doi:10.1108/jopp-10-01-2010-b002.

[32] Imperial MT . Using collaboration as a governance strategy. Adm Soc. 2005;37(3):281-320. doi:10.1177/0095399705276111.

[33] Jamali D . Success and failure mechanisms of public private partnerships (PPPs) in developing countries. Insights from the Lebanese context. Int J Public Sector Manag. 2004;17(5):414-430. doi:10.1108/09513550410546598.

[34] Kincaid J Stenberg CW . Big questions” about intergovernmental relations and management: who will address them? Public Adm Rev. 2011;71(2):196-202. doi:10.1111/j.1540-6210.2011.02330.x.

[35] Koppenjan JFM Enserink B . Public-private partnerships in urban infrastructures: reconciling private sector participation and sustainability. Public Administration Review. 2009;69(2):284-296. doi:10.1111/j.1540-6210.2008.01974.x.

[36] Krueathep W Riccucci NM Suwanmala C . Why do agencies work together? the determinants of network formation at the subnational level of government in Thailand. J Public Adm Res Theory. 2010;20(1):157-185. doi:10.1093/jopart/mun013.

[37] Martin MH Halachmi A . Public-private partnerships in global health: addressing issues of public accountability, risk management and governance. Public. Adm Q. 2012;36(2):189-212. https://www.researchgate.net/publication/289976663_Martin_M_H_and_Halachmi_A_2012_Public-private_partnerships_in_global_health_Addressing_issues_of_public_accountability_risk_management_and_governance_Public_Administration_Quarterly_362_189-212 (Accessed December 23, 2020).

[38] Minnery J . Inter-organisational approaches to regional growth management: a case study in South East Queensland. Town Plan Rev. 2001;72(1):25-44. https://www.jstor.org/stable/40111824?seq=1 (Accessed December 23, 2020).

[39] Ni AY . The risk-averting game of transport public-private partnership: lessons from the adventure of California’s state route 91 express lanes. Public Perform Manag Rev. 2012;36(2):253-274. doi:10.2753/PMR1530-9576360205.

[40] Oronje DO Rambo CM Odundo PA . Agency level management of roads maintenance levy fund: evidence from Kenya. Glob J Bus Res. 2014;8(1):73-85.

[41] Radin BA . Rural development councils: an intergovernmental coordination experiment. Publius: J Federalism. 1992;22(3):111-127. doi:10.1093/oxfordjournals.pubjof.a038014.

[42] Sagalyn L . Public-private partnerships and urban governance: coordinates and policy issues | Columbia business school research archive. In: Global Urbanization; 2011. (Accessed December 23, 2020). https://www8.gsb.columbia.edu/researcharchive/articles/5505

[43] Schaeffer Pv Loveridge S . Toward an understanding of types of public-private cooperation. Public Perform Manag Rev. 2002;26(2):169-189. doi:10.1177/1530957602238261.

[44] Silvestre HC de Araújo JFFE . Public-private partnerships/private finance initiatives in Portugal: theory, practice, and results. Public Perform Manag Rev. 2012;36(2):316-339. doi:10.2753/PMR1530-9576360208.

[45] Sundberg L Carlén G . Allocation mechanisms in public provision of transport and communication infrastructure. Ann Reg Sci. 1989;23(4):311-327. doi:10.1007/BF01579782.

[46] Tait M Hansen CJ . Trust and governance in regional planning. Town Plan Rev. 2013;84(3):283-312. https://www.jstor.org/stable/23474316?seq=1 (Accessed December 23, 2020).

[47] Torres C Briceño-Garmendia CM Dominguez C . Senegal’s infrastructure: a continental perspective. In: Policy Research Working Papers. Africa Region: The World Bank; 2011. doi:10.1596/1813-9450-5817.

[48] Wong C Webb B . Planning for infrastructure: challenges to Northern England. Town Plan Rev. 2014;85(6):683-708. doi:10.3828/tpr.2014.42.

[49] Malterud K Siersma VD Guassora AD . Sample size in qualitative interview studies: guided by information power. Qual Health Res. 2016;26(13):1753-1760. doi:10.1177/1049732315617444. 26613970

[50] Vaismoradi M Jones J Turunen H Snelgrove S . Theme development in qualitative content analysis and thematic analysis. Journal Nurs Educ Pract. 2016;6(5):100-110. doi:10.5430/jnep.v6n5p100.

[51] Al-Ghetaa RK Imtiaz D Shaw J Klein D Brown A . Supplemental material: the determinants of effective inter-organization information sharing in the health capital planning process - methods. doi:10.31219/OSF.IO/KBT7Y PMC9234776 35507410

